# NF-**κ**B Mediated Regulation of Adult Hippocampal Neurogenesis: Relevance to Mood Disorders and Antidepressant Activity

**DOI:** 10.1155/2014/612798

**Published:** 2014-02-12

**Authors:** Valeria Bortolotto, Bruna Cuccurazzu, Pier Luigi Canonico, Mariagrazia Grilli

**Affiliations:** Laboratory of Neuroplasticity, Department of Pharmaceutical Sciences, University of Piemonte Orientale, Via Bovio 6, 28100 Novara, Italy

## Abstract

Adult hippocampal neurogenesis is a peculiar form of process of neuroplasticity that in recent years has gained great attention for its potential implication in cognition and in emotional behavior in physiological conditions. Moreover, a vast array of experimental studies suggested that adult hippocampal neurogenesis may be altered in various neuropsychiatric disorders, including major depression, where its disregulation may contribute to cognitive impairment and/or emotional aspects associated with those diseases. An intriguing area of interest is the potential influence of drugs on adult neurogenesis. In particular, several psychoactive drugs, including antidepressants, were shown to positively modulate adult hippocampal neurogenesis. Among molecules which could regulate adult hippocampal neurogenesis the NF-**κ**B family of transcription factors has been receiving particular attention from our and other laboratories. Herein we review recent data supporting the involvement of NF-**κ**B signaling pathways in the regulation of adult neurogenesis and in the effects of drugs that are endowed with proneurogenic and antidepressant activity. The potential implications of these findings on our current understanding of the process of adult neurogenesis in physiological and pathological conditions and on the search for novel antidepressants are also discussed.

## 1. Introduction

The formation of new neurons has been demonstrated to continue throughout life in the adult brain of mammalians, including humans [1–9]. This process, referred to as adult neurogenesis, occurs in two restricted areas of the central nervous system (CNS), the subventricular zone (SVZ) in the lateral wall of the lateral ventricle, and the subgranular zone (SGZ) in the dentate gyrus (DG) within the hippocampal formation. In such regions, an instructive and permissive microenvironment, the so-called neurogenic niche, houses adult neural stem cells (aNSC) and functionally controls their development *in vivo*. Characteristics of aNSC are long-term self-renewal capacity and multipotentiality as they differentiate into multiple cell phenotypes, including neurons. In particular, in the hippocampal SGZ aNSC generate, by asymmetric division, precursors cells which migrate in the granular layer and, if they survive, differentiate into neurons that fully integrate into the preexisting functional network [4–8]. Altogether, adult neurogenesis is a complex process resulting from a fine balance between cell proliferation and cell death, migration, and differentiation and regulated by multifaceted molecular pathways.

In hippocampus adult neurogenesis is believed to be an important form of neural plasticity, enabling organisms to adapt to environmental changes and possibly influencing learning and memory throughout life. Indeed the process is highly modulable by external factors. Hippocampal neurogenesis is promoted by environmental enrichment and physical exercise and in turn increased hippocampal neurogenesis has been correlated with enhanced long-term potentiation in the dentate gyrus and improved spatial learning [9–13]. On the contrary, stress and aging reduce hippocampal neurogenesis and this reduction has been proposed to contribute to cognitive impairment observed in these settings [14–19].

A vast array of experimental studies suggested that adult hippocampal neurogenesis may be altered in various neuropsychiatric and neurodegenerative disorders [20–25], where its disregulation may contribute to cognitive impairment and/or emotional aspects of those diseases.

Another intriguing area of interest is the potential influence of drugs on adult neurogenesis. In particular, several psychoactive drugs were shown to be able to modulate either positively (antidepressants, atypical antipsychotics, and mood stabilizers) [26–30] or negatively (opioids, alcohol) adult neurogenesis [31–33].

Experimental work aimed at increasing our current knowledge on the molecular determinants of adult neurogenesis in physiological and pathological conditions holds the potential to help identifying novel therapeutic strategies aimed at targeting the endogenous pool of aNSC and their progeny in several neuropsychiatric disorders where neuroplasticity may be disregulated, including major depression disorder (MDD).

## 2. The Pleiotropic NF-**κ**B Family of Transcription Factors

In 1986, Baltimore and colleagues first described a B lymphocyte nuclear protein binding a 10 bp sequence in the enhancer region of the *κ* immunoglobulin light chain gene [34] and named that protein NF-*κ*B (nuclear factor in the kappa light chain enhancer of B cells). Since then, many researchers working in different fields have contributed to the idea that NF-*κ*B proteins represent a complex family of ubiquitously expressed transcription factors responsible for regulated expression of genes involved in pleiotropic functions, ranging from immunity and host defence to apoptosis, cell survival, cellular growth and repair, oncogenesis, and embryonic pattering [35, 36]. In mammals, the NF-*κ*B family consists of five structurally related proteins (p50 (NF-*κ*B1), p52 (NF-*κ*B2), p65 (RelA), c-Rel, and RelB), which all share the presence of the Rel homology domain responsible for DNA binding, homo- and heterodimerization, and interaction with the inhibitor of *κ*B (I*κ*B) protein and nuclear localization [37]. Additionally, p50 and p52 members are synthesized by proteolysis of their large precursors, named p105 and p100, respectively. According to their structure NF-*κ*B family members can be divided into two subfamilies: one includes RelA, RelB, and c-Rel, which contain a transactivator domain (TAD) and for this reason are considered transcriptional activators; the other one includes p50 and p52, which are generally considered as repressors, since lacking TAD. Family members are able to form homo- and heterodimers whose different compositions are responsible for multiple, sometimes even opposite, functions within the same cell type [38, 39]. At the cellular level, inactive NF-*κ*B dimers are retained into the cytoplasm by interaction with I*κ*B proteins [40], allowing a rapid activation in response to proper stimuli. A vast array of different stimuli are able to trigger NF-*κ*B by two distinct activating pathways, referred to as the canonical (classical) and the noncanonical (alternative) NF-*κ*B pathway. Canonical signaling relies upon I*κ*B kinase (IKK)-mediated degradation of I*κ*B, while the noncanonical signaling critically depends on NF-*κ*B inducing kinase (NIK)-mediated processing of p100 into p52. In the canonical pathway, I*κ*B kinase (IKK) phosphorylates Ser32 and Ser36 of the I*κ*B*α* subunit. Thereafter, the inhibitory protein is polyubiquitinated and degradated by 26S proteasome, with subsequent release of NF-*κ*B dimers [41]. In the noncanonical NF-*κ*B pathway, RelB/p100 complexes are inactive in the cytoplasm. Upon ligand stimulation, receptors such as LT*β*R, BR3, CD40, and receptor activator of nuclear factor kappa B (RANK) activate the NF-*κ*B inducing kinase (NIK) leading to the activation of IKK*α* homodimer (lacking IKK*γ*). Both NIK and IKK*α* phosphorylate p100. Phosphorylation of p100 leads to its ubiquitination and partial proteasomal processing into mature p52 subunits, resulting in transcriptionally competent RelB/p52 complexes that translocate to the nucleus and regulate a distinct class of genes [42].

To add an additional level of complexity and regulation within the system, NF-*κ*B activity is also regulated by post-translational changes, such as phosphorylation or acetylation on specific residues, which in turn can influence transcriptional activity, target gene specificity, or even termination of NF-*κ*B response [43]. Of particular interest is acetylation of RelA, which can occur at multiple lysines. Site-specific acetylation of p65 regulates discrete biological actions of the NF-*κ*B complex. It is of interest that acetylation of p65 on lysine 310 markedly enhances NF-*κ*B transactivation of some target genes [44, 45]. In the CNS, RelA Lys310 acetylation has been associated with both proapoptotic responses to ischemia [46] and with the analgesic effect of acetyl-*L*-carnitine in primary sensory neurons [47].

## 3. A Crucial Role for NF-**κ**B Transcription Factors in Postnatal Neurogenesis

In the CNS NF-*κ*B is expressed by both neuronal and nonneuronal cells, with p50/p65 and p50/p50 dimers representing the most abundant forms. Interestingly, in neuronal cells I*κ*B-complexed NF-*κ*B dimers are also present in synapses [48–54], suggesting that in this cell type NF-*κ*B proteins not only act as transcriptional regulators but they may represent crucial synapse-to-nucleus messengers. Interestingly, various forms of synaptic plasticity can regulate NF-*κ*B subcellular distribution, DNA binding activity, and transcription [51, 53, 55–60].

Within CNS, activation of NF-*κ*B has been involved in cell differentiation and survival [61, 62], proliferation and migration, and cell death programs [63, 64]. More recently, emerging data demonstrated its crucial involvement in regulating the growth and complexity of neuronal arborizations [65–69] and in synaptic plasticity and memory in the adult brain [56, 70–72]. Based on the widespread role of this protein family, it is not at all surprising that NF-*κ*B-mediated transcriptional programs could also be involved in the translation of the complex and integrated signals which regulate adult neurogenesis.

A few years ago, accidentally, we discovered that NF-*κ*B family members are expressed at considerable levels in neurogenic areas of postnatal and adult mouse brain [73] and based on that initial observation we proposed that they may be involved in the regulation of adult neurogenesis. Since then, a vast array of information has been collected on the complex involvement of NF-*κ*B proteins in different aspects of postnatal neurogenesis. Several extracellular signals have been identified as being able to affect NSC and/or their progeny via NF-*κ*B activation. The cytokine tumour necrosis factor *α* (TNF-*α*) was identified as an *in vitro* inducer of adult rat neural stem cell proliferation via NF-*κ*B, as confirmed by pharmacological blockade with a transdominant negative superrepressor I*κ*B*α*-dn and by IKK*β* knock-down [74]. In line with these reports, others have demonstrated in SVZ-derived neurospheres the presence of TNF-R1, whose engagement resulted in activation of NF-*κ*B and increased neural stem cell proliferation and neuronal differentiation [75]. Erythropoietin was also shown to act as a homeostatic autocrine/paracrine signaling molecule that would direct multipotent NSC to become neural progenitors by activating nuclear translocation of NF-*κ*B [76]. More recently, Wada and colleagues [77] demonstrated that vascular endothelial growth factor (VEGF) via its flk1 receptor directly promotes adult NSC survival and that these effects are mediated by NF-*κ*B. Glutamate is also a well-described activator of NF-*κ*B [50]. Brazel and colleagues reported that the neurotransmitter enhances survival and triggers proliferation of SVZ-derived NSC [78]. In 2007 the group of Michal Schwartz demonstrated that Toll-like receptors (TLR) are expressed by adult neural stem/progenitor cells where they play distinct and opposite functions in NSC proliferation and differentiation both *in vitro* and *in vivo* [79]. Indeed TLR2 deficiency in mice impaired hippocampal neurogenesis, whereas the absence of TLR4 resulted in enhanced proliferation and neuronal differentiation [79]. The activation of TLRs on NSCs was mediated via MyD88 and protein kinase C (PKC) *α*/*β*-dependent activation of the NF-*κ*B signaling pathway [79]. Also neural stem/progenitor cell migration can be regulated by NF-*κ*B proteins. As an example, among NF-*κ*B target genes, monocyte chemoattractant protein-1 (MCP-1) is able to stimulate migration of adult rat NSC by interaction with the chemochine (C-C motif) receptor 2 (CCR2) [80].

Among upstream receptors which could activate NF-*κ*B signaling pathways and in turn affect neurogenesis we have recently proposed the receptor for advanced glycation end-products (RAGE). We indeed demonstrated RAGE expression in undifferentiated neural stem/progenitor cells of mouse adult hippocampal and SVZ neurogenic regions [81, 82]. Several RAGE ligands, including the alarmin HMGB-1, S100*β*, and AGE-BSA, stimulated both proliferation and neuronal differentiation of SVZ-derived NPC *in vitro*. NF-*κ*B nuclear translocation occurred upon RAGE activation in SVZ-derived neurospheres and its blockade (by SN-50) or its absence (in p50^−/−^ derived NPC) resulted in the inhibition of the ligand-mediated effects on neuronal differentiation [81]. More recently, we have been able to show that the alarmin HMGB-1 and A*β*_1−42_ oligomers, both involved in AD pathophysiology [83, 84], can promote neuronal diffe micerentiation of adult hippocampal NPC and that their proneurogenic activity is mediated by activation of RAGE/NF-*κ*B axis [82].

Our group extensively investigated the role of the NF-*κ*B p50 subunit in adult hippocampal neurogenesis, by taking advantage of p50^−/−^ mice generated in the laboratory of David Baltimore [85]. In this genetically modified animal model we could demonstrate a marked deficiency in the number of new neurons generated in the hippocampi of p50^−/−^ mice [86]. Interestingly, the proliferation rate of neural stem cells in the SGZ of p50 deficient mice appeared to be similar to that of wt mice. In contrast, the survival rate of BrdU labeled cells at 21 days after thymidine analog administration was remarkably reduced in mutant compared to wt mice. A detailed phenotypic characterization of newly generated cells revealed no difference in the number of doublecortin (DCX) positive neuroblasts but a marked reduction of calretinin (CR)^+^ postmitotic neurons in the DG of mutant mice, compared to the wt counterpart. Based on these findings we proposed that absence of the NF-*κ*B p50 subunit may trigger a selective defect in adult neurogenesis progression at the transition between the maturation stages of newly born neuroblasts and postmitotic neurons characterized by the expression of DCX and CR, respectively [86]. To test the physiological consequences of alterations in hippocampal neurogenesis, wt and p50^−/−^ mice were also evaluated at the behavioural level. When tested in the Morris water maze, wt and p50^−/−^ mice performed equally well in the acquisition test. Similarly, in the probe test, all mice, regardless of the presence or the absence of NF-*κ*B p50, showed a target quadrant preference and an increased time spent in the target quadrant in search of the missing platform, suggesting normal retrieval of hippocampal-dependent long-term spatial memory in both genotypes. Mice were also subjected to the place recognition test that evaluates hippocampal-dependent short-term spatial memory by testing animal ability to discriminate a familiar versus a novel environment. Compared with wt mice, p50^−/−^ mice showed a selective impairment in short-term spatial memory. Altogether, p50^−/−^ mice not only exhibited specific deficits in net adult hippocampal neurogenesis but also an impairment in a hippocampal-dependent task of short-term spatial memory. Of course these data do not imply a cause-effect relationship between neurogenesis defects and selective deficits in short-term spatial memory exists in p50^−/−^ mice, but certainly the correlation between these phenomena deserves further investigation. Interestingly, other groups have demonstrated cognitive problems in p50^−/−^ mice [87]. In addition, treatment with SN50, a cyclic peptide that masks p50 NLS, can result in impaired memory reconsolidation in mice [88]. Interestingly, we have recently evaluated if any alteration was present, in absence of p50, in the SVZ neurogenic region. No defect could be observed in the olfactory bulb of p50^−/−^ mice compared to wt littermates, suggesting a hippocampus-specific effect of NF-*κ*B p50 absence (Bortolotto et al., *data not shown*). At present, it is not clear whether the phenotypic changes in p50^−/−^ mice depend on the derepression or on the lack of activation of specific target genes. Targeted disruption of the p50 gene should have profound consequences on the pool of dimeric NF-*κ*B complexes because p50 homodimers, generally considered as transcriptional repressors, but also p50/p65 heterodimers, as well as any other p50-containing heterodimer, which can act as activators, are not formed in mutant mice. It is very likely that the complex phenotype of p50^−/−^ mice, including impairment of short-term spatial memory but intact learning and long-term memory, may in part result from these complex changes.

Deficits in specific hippocampal-dependent cognitive tasks have been reported in knockout mice for other NF-*κ*B subunits [70, 89, 90], but no correlation has ever been drawn between those deficits and reduced adult neurogenesis. It would certainly be of great interest to investigate whether the deficits in cognitive performance of mouse lines genetically impaired in the NF-*κ*B signaling would correlate with abnormalities in hippocampal neurogenesis. Recently one research group presented evidence that NF-*κ*B ablated mice are characterized by a severe atrophy and by astrogliosis in the DG [91]. This condition was proposed to rely on the dual function of NF-*κ*B in the hippocampal region: in neural progenitor progeny NF-*κ*B could be involved in axogenesis and maturation, whereas in mature granule cells it regulates neuroprotection as well as synaptic transmission. When NF-*κ*B was inactivated, mossy fibers, the axons of granule cells, degenerated and addition of newborn neurons could not take place in the DG. Interestingly, reactivation of NF-*κ*B led to regrowing of the dentate gyrus and recovery of structural defects by reexpression of the downstream targets FOXO1 and PKA, responsible for axonal outgrowth and axon fate determination, respectively [91].

In parallel with data demonstrating the important role of NF-*κ*B-mediated transcriptional programs in promoting adult neuroplasticity, additional evidence support the idea that sustained activation of NF-*κ*B in neurons may have negative consequences on hippocampal integrity and cognitive performance. By using a conditional gain-of-function mouse model that expresses a constitutive allele of IKK*β* in forebrain neurons, Maqbool and colleagues [92] demonstrated that persistent chronic IKK*β*/NF-*κ*B activation induces selective inflammatory response in the DG, associated with decreased neuronal survival and severe cognitive impairment. Although neurogenesis was not directly investigated in that study, it is noteworthy that the observed DG changes were also paralleled by downregulation of hippocampal BDNF levels, whose contribution to regulation of adult neurogenesis is well established. In a very elegant manner the same authors demonstrated that neuronal loss in DG was restored when chronic IKK*β*/NF-*κ*B activation was turned off and BDNF levels were restored.

The role of NF-*κ*B signaling in regulating different steps in the neurogenesis process (stem/progenitor cell proliferation, neuroblast differentiation, migration, maturation, and integration of postmitotic neurons) certainly deserves further analysis. In NF-*κ*B p50^−/−^ mice our group reported no alteration in the proliferation rate of hippocampal NPC but rather a defect in late maturation of neuroblasts in postmitotic neurons, compared to wt littermates [73]. In a different context, Zhang et al. [93] proposed a crucial role of NF-*κ*B signaling in the regulation of very early stages of neurogenesis. Although these authors utilized adult NSC/NPC from SVZ rather than from hippocampus, their work suggested that the NF-*κ*B pathway may be inactive in proliferating nestin^+^/sox2^+^ NSC, while it is activated only after growth factor removal which triggers differentiation toward neuronal, astroglial, and oligodendroglial lineages. Moreover they showed that inhibition of NF-*κ*B activation, either pharmacologically (by shRNA) or genetically (by using a transgenic mouse line expressing dnI*κ*B*α* driven by the GFAP promoter [94]), would result in blockade of asymmetric division and neural differentiation at a very early stage, so leading to accumulation of NSC. Furthermore C/EBP*β* was identified as an effector of NF-*κ*B-mediated early differentiation of NSC/NPC.

Although it is not the focus of this review, the complex role of NF-*κ*B signaling pathways in the regulation of adult neurogenesis may not be limited to DG and SVZ, but also to more recently identified areas where NSC reside, including the hypothalamus. In such region adult hypothalamic NSC (htNSC) appear to be important for central regulation of metabolic physiology, including feeding, body weight, and glucose homeostasis [95, 96]. Interestingly, several research groups revealed that activation of the IKK*β*/NF-*κ*B pathway mediated high-fat diet induced hypothalamic inflammation to cause metabolic syndrome [97, 98]. More recently, mouse studies revealed that hypothalamus-specific IKK*β*/NF-*κ*B activation led to depletion and impaired neuronal differentiation of htNSC and ultimately to obesity and prediabetes development [99].

## 4. Adult Hippocampal Neurogenesis and Major Depressive Disorder

Although the role of adult hippocampal neurogenesis remains yet to be fully elucidated, the possibility that the process is involved in cognitive and emotional functions [100–103] and deregulated in various neuropsychiatric disorders, including MDD [25, 104, 105], has been proposed.

Based on the evidence that hippocampal neurogenesis can be downregulated under stressful conditions, including those that result in animal models of depressive-like behaviors, and it can be upregulated by antidepressant drugs and treatments, the hypothesis has emerged that neurogenesis, together with other related aspects of hippocampal plasticity, may contribute to the pathophysiology of MDD and its effective treatment [106–108].

In particular, several authors have suggested that neurogenesis may be necessary for some, although not all, of the behavioural effects of antidepressants. The induction of adult neurogenesis has been observed after chronic treatment with antidepressant drugs with different mechanisms of action, such as selective serotonin reuptake inhibitors (SSRI), tricyclic antidepressants (TCA), and monoamine oxidase inhibitors (IMAO) [109, 110]. In particular, it has been demonstrated that chronic, but not acute, treatment with monoaminergic antidepressants significantly increased the number of BrdU positive cells in the DG of treated animals compared to vehicle, when animals are killed two hours after BrdU administration [109]. These data indicated that in the hippocampus, cell proliferation was increased after chronic antidepressants, in a manner which is consistent with the time course for therapeutic action of antidepressants [111]. Moreover, Santarelli and colleagues [110] induced ablation of hippocampal adult neurogenesis using cranial X-irradiation and found that irradiated mice no longer responded behaviorally to fluoxetine, suggesting that enhanced hippocampal neurogenesis may be required for antidepressant activity. The proneurogenic effects of antidepressants are not restricted to drugs increasing monoaminergic neurotransmission. Chronic treatment with CRF-R1 and V1b receptor antagonists, which are endowed with antidepressant-like properties in predictive animal models [112, 113], also exerted a positive influence on hippocampal granule cell proliferation, reversing negative effects elicited by chronic mild stress [114]. Also the recently introduced antidepressant agomelatine promotes hippocampal neurogenesis. Chronic administration of this MT1/MT2 melatonin receptor agonist and 5-HT_2B/2C_ receptor antagonist significantly increased the number of newborn cells in the hippocampus of adult rats [115]. Additionally, the drug reversed defective hippocampal neurogenesis caused in adulthood by prenatal stress [116]. Recently, David et al. [117] utilizing the mouse model of anxiety/depressive-like state induced by chronic corticosterone treatment contributed to a better understanding of the link between antidepressants and hippocampal neurogenesis. When neurogenesis was ablated in these mice by hippocampal X-irradiation, the efficacy of fluoxetine was blocked in some, but not all, behavioral paradigms, suggesting the existence of both neurogenesis-dependent and -independent mechanisms of antidepressant action. Altogether currently available data are compatible with the idea that antidepressant treatments commonly share the capacity to positively modulate hippocampal neurogenesis. Recently the evidence that antidepressants may act on the endogenous pool of aNSC has been extended also to humans. Postmortem brain tissue of MDD subjects either untreated or treated with SSRI (sertraline, fluoxetine) and TCA (nortriptyline, clomipramine) antidepressants were analyzed by Boldrini and colleagues [118]. Quantification of NPC (nestin^+^ cells) and dividing cells (Ki-67^+^ cells) in the DG confirmed a significant increase in the number of nestin^+^ and Ki-67^+^ cells in treated—compared with untreated—MDD patients and with age-matched controls [118].

Altogether, the current state of knowledge allows to propose adult hippocampal neurogenesis as a potential substrate underlying antidepressant therapeutic effects.

## 5. NF-**κ**B Signaling Pathway as a Converging Mechanism for Drugs Affecting Neurogenesis and Exerting Antidepressant Activity 

During the past five years, we have been actively searching for new molecules endowed with the potential to regulate *in vitro* and *in vivo* hippocampal neurogenesis and asked whether this property could correlate with an antidepressant effect. The ongoing search for new antidepressants is justified by the fact that several limitations are associated with the currently available ones, including the fact that drugs have a delayed onset of action (6–8 weeks) and in many cases they have considerable side effects that limit their use in subpopulations like elderly patients. Last but not least, the high number of patients that are resistant to treatment represents an important challenge in the clinical setting.

Herein we would like to share recent findings on two drugs which are already available in clinic and that we propose as novel positive modulators of hippocampal neurogenesis and as a potential new antidepressant drugs. Incidentally, both drugs require NF-*κ*B activation for their proneurogenic and antidepressant effects in animal models.

One first example is represented by the so-called gabapentinoids pregabalin (PGB) and gabapentin (GBP). These drugs are clinically relevant anticonvulsant, analgesic, and anxiolytic drugs [119, 120]. A large collection of data has contributed to the idea that their therapeutic effects would rely on their ability to bind the *α*2*δ*1 subunit of neuronal voltage-gated calcium channels [121, 122]. Interestingly, the literature reports have also suggested that these drugs may be beneficial, when given as add-on during antidepressant therapy, on depression-like symptoms in patients affected by posttraumatic stress [123] and generalized anxiety disorders [124], with an unknown mechanism. We recently demonstrated that adult hippocampal neural progenitors do express the *α*2*δ*1 subunit [125]. While investigating the potential activity of several anticonvulsant drugs in an *in vitro* mouse model of adult neural progenitor cells, we discovered that only GBP and PGB resulted in a concentration-dependent positive effect on NPC differentiation toward the neuronal lineage. *In vivo* studies confirmed that chronic administration of PGB increased the number of newly generated neurons in the dentate gyrus of adult mice, without affecting their rate of proliferation or survival [125]. *In vitro*, the *α*2*δ* antagonists *L*-isoleucine and *L*-(+)-*α*-phenylglycine inhibited PGB-induced proneurogenic effects, suggesting that they were mediated by interaction with the *α*2*δ*1 subunit. Interestingly, activation of the NF-*κ*B pathway was involved in the proneurogenic effects elicited by *α*2*δ* ligands in adult hippocampal NPC, because inhibition of both p50 and p65 nuclear translocation and IKK*β* counteracted PGB-mediated effects. Moreover, the proneurogenic effects of pregabalin were also ablated in aNPC from p50^−/−^ mice (*data not shown*). Last but not least, we demonstrated that chronic PGB administration prevented the appearance of depressive-like behaviour induced by chronic restraint stress and, in parallel, promoted hippocampal neurogenesis in adult stressed mice. Altogether, these data allowed us to propose, for the first time, a novel pharmacological property of *α*2*δ* ligands, namely, positive modulation of adult neurogenesis. Whether the proneurogenic activity of *α*2*δ* ligands, via NF-*κ*B activation, may contribute to drug efficacy on depressive symptoms in patients certainly deserves further investigation, since the dose of PGB in our studies yields plasma concentrations comparable with effective dosage in clinical practice [122, 126].

Another drug that we recently proposed to have proneurogenic and antidepressant activity via modulation of NF-*κ*B signaling pathway is acetyl-*L*-carnitine (ALC). Endogenous ALC, aside from its role in cellular bioenergetics, can act as a donor of acetyl groups to proteins [127], including NF-*κ*B p65 [47]. Exogenously administered ALC can readily pass the blood-brain barrier [128] and it is neuroprotective at supraphysiological concentrations [129]. In addition, the antinociceptive effects of ALC were demonstrated in rodent pain models [47, 130]. In humans, and particularly in the elderly, the beneficial effects of ALC were reported in mood disorders [131–134], with a totally unknown mechanism of action. Surprisingly, no published studies have evaluated, in animal models, the antidepressant activity of ALC and the putative mechanism involved in such effect. Interestingly, ALC-mediated modulation of metabotropic glutamate receptor 2 (mGlu2) gene expression via NF-*κ*B p65 acetylation has been proposed as the mechanism underlying for ALC analgesic effects [47, 130]. We therefore explored the possibility that ALC may promote hippocampal neurogenesis. As reported in Cuccurazzu et al. [135] ALC proved to be a potent proneurogenic molecule, whose effect on neuronal differentiation of adult hippocampal neural progenitors is independent of its neuroprotective activity. The *in vitro* proneurogenic effects of ALC appear to be mediated by activation of the NF-*κ*B pathway and subsequent NF-*κ*B-mediated upregulation of metabotropic glutamate receptor 2 (mGlu2) expression. More specifically, ALC resulted in acetylation of p65 at Lys(310) in adult hippocampal NPC cultures. Moreover, ALC treatment of hippocampal NPC resulted in a significant upregulation of mGlu2 protein levels and this effect was abolished by inhibiting p65 nuclear translocation. When tested *in vivo*, chronic ALC treatment could revert depressive-like behavior caused by unpredictable chronic mild stress, a rodent model of depression with high face validity and predictivity, and its behavioral effect correlated with upregulated expression of mGlu2 receptor in hippocampi of stressed mice. Moreover, chronic, but not acute or subchronic, drug treatment significantly increased adult born neurons in mouse hippocampi. A paper by Nasca et al. [136] confirmed and further extended our results. These authors demonstrated ALC-mediated antidepressant effects in a genetic model of depression, the Flinders Sensitive Line rats [137]. Even in their setting, the drug increased acetylation of NF-*κ*B-p65 subunit, thereby enhancing the transcription of mGlu2 receptor in hippocampus and prefrontal cortex. It is particularly interesting that in their models the authors compared ALC and chlorimipramine, a tricyclic antidepressant, and came to the conclusion that ALC reduced the immobility time in the forced swim test and increased sucrose preference as early as 3 days of treatment, whereas 14 days of treatment were needed for the antidepressant effect of chlorimipramine. Moreover, ALC antidepressant effects were still present two weeks after drug withdrawal. The rapid and long-lasting antidepressant action of ALC strongly suggested a unique mode of action for this drug which deserve further investigation, since potentially paving the way for more efficient antidepressants with faster onset of action. Altogether our and Nasca's data propose a novel mechanism, involving mGlu2 receptor upregulation and hippocampal neurogenesis, via NF-*κ*B p65 acetylation, which could explain the antidepressant effect of ALC in humans. From a clinical perspective these results are relevant also in view of ALC high tolerability profile which could allow to employ the drug in patient subpopulations who are sensitive to the side effects associated with classical monoaminergic antidepressants.

Incidentally NF-*κ*B-mediated transcription appeared to be involved in both proneurogenic and antidepressant effects of both ALC and *α*2*δ*1 ligands. Of course we do not intend to propose that NF-*κ*B activation may represent itself a target for antidepressant drugs. Ja and Duman [138] have demonstrated an essential role of the proinflammatory cytokine interleukin-1 beta (I*L*-1*β*) in the antineurogenic effects of chronic stress, a condition which represents a major risk factor for depressive disorders. By *in vivo* and *in vitro* studies these authors provided evidence that adult hippocampal progenitor cells do indeed express I*L*-1*β* receptor and that its activation decreases cell proliferation via the NF-*κ*B signaling pathway. Indeed, in that experimental setting inhibitors of NF-*κ*B/IKK signaling significantly blocked the antineurogenic effects of I*L*-1*β* in adult hippocampal progenitors [138]. The fact that both induction and inhibition of adult neurogenesis may rely on NF-*κ*B p65 is likely to reflect the complexity within the NF-*κ*B signaling pathway. NF-*κ*B proteins represent a family whose members, including p65, can undergo different posttranslational modifications and can combine to form dimers of different composition, which can be differentially activate and exert different, even opposite, functions through activation of different sets of gene targets in a given cell type [139]. To this regard, too little information is currently available on the contribution of different p65 posttranslational modifications to distinct NF-*κ*B-mediated transcriptional program in the CNS. In the future it will be important to identify the full set of NF-*κ*B gene targets activated in the hippocampus by proneurogenic and antidepressant drugs whose products, like mGlu2, may represent novel therapeutic targets.

## 6. Conclusions

The literature data herein summarized support the idea that the NF-*κ*B signalling pathway may play an important regulatory role in adult hippocampal neurogenesis both in physiological and pathophysiological conditions. Furthermore these data allow to propose that NF-*κ*B signalling may also be potentially involved in mediating the proneurogenic and antidepressive-like activity of some clinically relevant drugs. Whether the proneurogenic activity of *α*2*δ* ligands and ALC, via NF-*κ*B activation, may contribute to their efficacy on depressive symptoms in patients still deserves further investigation. Although still limited, these data also point to the relevance of identifying the full set of NF-*κ*B target genes downstream these proneurogenic and antidepressant molecules, since their encoding products may represent potential targets for novel therapeutic strategies in depressive disorders.
